# QTL Mapping by SLAF-seq and Expression Analysis of Candidate Genes for Aphid Resistance in Cucumber

**DOI:** 10.3389/fpls.2016.01000

**Published:** 2016-07-11

**Authors:** Danna Liang, Minyang Chen, Xiaohua Qi, Qiang Xu, Fucai Zhou, Xuehao Chen

**Affiliations:** ^1^School of Horticulture and Plant Protection, Yangzhou UniversityYangzhou, China; ^2^Sericulture/Chili Pepper Research Institute, Guizhou Academy of Agricultural SciencesGuiyang, China

**Keywords:** cucumber, aphid resistance, SLAF-seq, QTL, candidate genes

## Abstract

Cucumber, a very important vegetable crop worldwide, is easily damaged by pests. Aphid is one of the most serious cucumber pests and frequently cause severe damage to commercially produced crops. Understanding the genetic mechanisms underlying pest resistance is important for aphid-resistant cucumber varieties breeding. In this study, two parental cucumber lines, JY30 (aphid susceptible) and EP6392 (aphid resistant), and pools of resistant and susceptible (*n* = 50 each) plants from 1000 F_2_ individuals derived from crossing JY30 with EP6392, were used to detect genomic regions associated with aphid resistance in cucumbers. The analysis was performed using specific length amplified fragment sequencing (SLAF-seq), bulked segregant analysis (BSA), and single nucleotide polymorphism index (SNP-index) methods. A main effect QTL (quantitative trait locus) of 0.31 Mb on Chr5, including 43 genes, was identified by association analysis. Sixteen of the 43 genes were identified as potentially associated with aphid resistance through gene annotation analysis. The effect of aphid infestation on the expression of these candidate genes screened by SLAF-seq was investigated in EP6392 plants by qRT-PCR. The results indicated that seven genes including encoding transcription factor MYB59-like (Csa5M641610.1), auxin transport protein BIG-like (Csa5M642140.1), F-box/kelch-repeat protein At5g15710-like (Csa5M642160.1), transcription factor HBP-1a-like (Csa5M642710.1), beta-glucan-binding protein (Csa5M643380.1), endo-1,3(4)-beta-glucanase 1-like (Csa5M643880.1), and proline-rich receptor-like protein kinase PERK10-like (Csa5M643900.1), out of the 16 genes were down regulated after aphid infestation, whereas 5 genes including encoding probable leucine-rich repeat (LRR) receptor-like serine/threonine-protein kinase At5g15730-like (Csa5M642150.1), Stress-induced protein KIN2 (Csa5M643240.1 and Csa5M643260.1), F-box family protein (Csa5M643280.1), F-box/kelch-repeat protein (Csa5M643290.1), were up-regulated after aphid infestation. The gene Csa5M642150.1, encoding probable LRR receptor-like serine/threonine-protein kinase At5g15730-like, was most likely a key candidate gene in cucumber plants in response to infestation. This study provides a certain theoretical basis of molecular biology for genetic improvement of cucumber aphid resistance and aphid resistant variety breeding.

## Introduction

Cucumber, *Cucumis sativus* L. (2*n* = 2x = 14), is an agriculturally and economically important vegetable crop worldwide. The aphid, *Aphis gossypii* Glover, is found in the majority of cucumber producing areas in China and is a serious pest, often causing severe yield loss and reduced quality in cucumber production. Understanding the genetic mechanisms underlying aphid resistance is important for breeding aphid resistance and improving cucumber quality.

Determination of the genetic basis of crop resistance to insect pests has been widely studied. Investigation of sorghum resistance to the greenbug using different resistant plant lines and various aphid biotypes, revealed 3 to 9 associated genomic regions (Agrama et al., 2002; Wu and Huang, 2008). Quantitative trait loci (QTLs) identified as associated with aphid resistance include: five in barley, conferring resistance to the Russian wheat aphid (Mittal et al., 2008); two in cowpea, controlling soybean aphid resistance (Zhang et al., 2009); four additive and two couples of epistatic QTLs in melon, responsive to the melon-cotton aphid infestation (Boissot et al., 2010); and one major candidate QTL on chromosome 7 controlling foxglove aphid resistance in soybean (Lee et al., 2015). In addition, a putative QTL in apple for resistance to the rosy apple aphid and another for resistance to the green apple aphid have been localized (Stoeckli et al., 2008), and in peach, two QTLs for resistance to the green peach aphid were identified (Sauge et al., 2004). However, the genetic mechanisms underlying aphid resistance in cucumber remain unclear.

In soybean, three dominant genes for resistance to the soybean aphid, *Rag1*, *Rag2*, and *Rag3*, have been mapped to independent soybean linkage groups (Hill et al., 2009; Zhang et al., 2010). In addition, previous studies have provided evidence that events mediated by E3 ubiquitin, auxin, and sugar changes in plants infested with aphids led to transcriptional regulation of responses to plant diseases and pests (Pieterse et al., 2009; Stegmann et al., 2012). Resistance (R) genes, encoding proteins containing leucine-rich repeat (LRR) regions such as: *Gb3* (Weng and Lazar, 2002; Weng et al., 2005; Azhaguvel et al., 2012), *Mi* (Milligan et al., 1998; Rossi et al., 1998; Goggin et al., 2001), and *Vat* (Pauquet et al., 2004; Boissot et al., 2010), among others, are major genes in aphid resistance.

Quantitative trait loci mapping is the main approach for genetic dissection of quantitative traits. It is conventionally conducted by genotyping a large number of individuals in segregating populations, which is labor-intensive, time-consuming and sometimes costly (Salvi and Tuberosa, 2005). Bulked-segregant analysis (BSA) provides a simple and effective alternative technology to identify molecular markers linked to target genes or QTLs affecting a trait of interest, by genotyping only one pair of pooled DNA samples from two groups of individuals with distinct or opposite extreme phenotypes (Michelmore et al., 1991). Specific length amplified fragment sequencing (SLAF-seq) combining with high-throughput and reduced representation library sequencing is considered an efficient and high-resolution strategy for large-scale genotyping (Sun et al., 2013). In maize, three candidate regions related to genetic and molecular control of meristems on Chr3, containing 51 candidate genes and consisting of 3.947 Mb, were obtained by SLAF-seq (Xia et al., 2015). In cucumber, a major QTL controlling fruit flesh thickness was identified by SLAF-seq and confirmed by simple sequence repeat marker-based classical QTL mapping in 138 F_2_ individuals (Xu et al., 2015).

In this study, pools of resistant and susceptible bulk samples (*n* = 50 each) were constructed from 1000 F_2_ plants derived from a cross of JY30 (susceptible female parent, P_1_) and EP6392 (resistant male parent, P_2_) plants, and were used to detect regions in the cucumber genome harboring major aphid resistance QTLs by SLAF-seq, BSA and single nucleotide polymorphism index (SNP-index) methods. The effect of infestations with different numbers of aphids on the expression of genes identified by SLAF-seq were studied by qRT-PCR.

## Materials and Methods

### Aphid Culture

One aphid (*Aphis gossypii* Glover) was collected from the experimental fields of cucumber at Yangzhou University in the autumn of 2012, and reared on the susceptible cucumber line ‘XiaFeng’ at 25°C (18 h)/18°C (6 h) day/night in relative humidity of 50–60%. The offspring of this aphid were used to infest cucumber plants.

### Plant Materials and Aphid Infestation

All plants were cultivated in potting substrate (nutrient availability: total N, P, and K nutrients, 40–60 g/kg; total humus content, ≥350 g/kg; pH 6.5–7.5) in environmental growth chambers maintained at 25°C (18 h)/18°C (6 h) day/night, at a light intensity of 12,000 lux (18 h)/0 lux (6 h), and relative humidity between 50 and 60%. An F_2_ population of 1000 individuals derived from a cross of JY30 (susceptible female parent, P_1_) and EP6392 (resistant male parent, P_2_) was used for genetic analysis and molecular mapping of aphid resistance QTLs. Both EP6392 and JY30 are European cucumber. The commercial fruit length of EP6392 is about 25-30 cm, while 30-35 cm of JY30. The fruit peel of EP6392 is dark green, less thorn, however, the fruit peel of JY30 is green, spiny. The leave of EP6392is dark green, while the leaves of JY30 is green. Seeds of P_1_, P_2_, and F_2_ generations were sown on 28 May, 2014. Ten days after sowing, five apterous adult aphids were transferred to the back of the first true leaf per seedling. The number of aphids on individual plants were counted on day 8 after infestation and recorded as aphid scores ranging from 1 to 5, where 1 ≤ 100 aphids, 2 = 101 to 200 aphids, 3 = 201 to 300 aphids, 4 = 301 to 400 aphids, and 5 ≥ 401 aphids per plant (scoring system was modified based on Jun et al., 2012).

The expression of genes identified by SLAF-seq after infestation of EP6392 host plants with 5 (T1) or 20 (T2) aphids per plant, or controls without aphids (CK), was determined on day 6 after infestation. Thirty seeds were sown on 6 June, 2015 and apterous adult aphids were transferred to the back of the first true leaf per seedling 10 days after sowing. Three replicate experiments, using three separate plants each replication, were conducted for each treatment (CK, T1, and T2).

### DNA Isolation and SLAF Library Construction for High-Throughput Sequencing

Leaves from the two parent plants (JY30 and EP6392) and F_2_ individuals were collected on day 8 after aphid infestation, and an equal amount (0.1 g per plant) of leaves from 50 resistant and 50 susceptible individuals from F_2_ plants were pooled, frozen in liquid nitrogen, and used for DNA extraction. Total genomic DNA was prepared using the CTAB method, with a modified CTAB buffer (8.18 g NaCl and 2 g CTAB in a total volume of 100 mL of 20 mM EDTA, 100 mM Tris, pH 8.0). DNA concentration and quality were estimated using a BioPhotometer Plus spectrophotometer (Eppendorf, Hamburg, Germany) and by electrophoresis through 1% agarose gels. Two DNA pools, resistant (R-pool) and susceptible (S-pool), were constructed using DNA isolated from pooled leaves from 50 individuals each of the 1000 F_2_ plants. DNA samples isolated from JY30 and EP6392 plant leaves, and the two DNA pools, were used for SLAF library construction and sequencing.

A pilot experiment was performed to establish the conditions required to achieve optimal SLAF yield, avoid repetitive SLAFs, and obtain an even distribution of SLAFs for maximum SLAF-seq efficiency. We constructed the SLAF library based on the result of the pilot experiment. Genomic DNA from each sample was incubated with HaeIII and RsaI, T4 DNA ligase, ATP, and an RsaI adapter (all from New England Biolabs (NEB), Ipswich, MA, USA) at 37°C. Then, the restriction-ligation reaction solutions were diluted and mixed with dNTPs, Taq DNA polymerase (NEB) and primers containing barcode 1 for polymerase chain reaction (PCR). An EZNA^®^ Cycle-Pure Kit (Omega, London, UK) was used to purify the PCR products, which were then pooled and incubated at 37°C with MseI (NEB), T4 DNA ligase, ATP, and a Solexa adapter. After incubation, the reaction products were purified using a Quick Spin column (Qiagen, Hilden, Germany) and electrophoresed through a 2% agarose gel. After gel purification, DNA fragments (SLAFs, including adapter sequence indices and adaptors) of 264–364 bp were excised and diluted for paired-end sequencing (Xu et al., 2015). SLAF-seq was performed using the Illumina HighSeq 2500 platform, with samples prepared according to the Illumina sample preparation guide (Illumina, Inc, San Diego, CA, USA) at the Biomarker Technologies Corporation (Beijing, China).

### Analysis of SLAF-seq Data

SNP_index is a marker association analysis method to determine differences in genotype frequencies between pooled samples (Abe et al., 2012), by calculation of the Δ(SNP_index). The stronger the correlation between a marker and a trait, the closer the Δ(SNP_index) value is to 1. In this study, ‘M,’ ‘P,’ ‘aa,’ and ‘ab’ denote the female parent (susceptible, JY30), the male parent (resistant, EP6392), the R-pool and the S-pool, respectively. Δ(SNP_index) values were calculated as follows: SNP_index(ab) = Mab/(Pab+Mab); SNP_index(aa) = Maa/(Paa+Maa); Δ(SNP_index) = SNP_index(aa) – SNP_index(ab); where Maa indicates the depth of the aa population derived from M, Paa indicates the depth of the aa population derived from P, Mab indicates the depth of the ab population derived from M, and Pab indicates the depth of the ab population derived from P. The allelic frequency was calculated by Euclidean distance followed by Loess regression analysis which identifies regions in which QTL lies and generates a list of putative regions in the linked genomic segment.

### RNA Isolation, Reverse Transcription, and qRT-PCR Analysis

Leaves from cucumber plants in CK, T1 and T2 treatments were collected on day 6 after aphid infestation, frozen in liquid nitrogen, and used for RNA extraction. Total RNA was isolated from leaves using TakaRa RNAiso Reagent kit (Dalian, China). RNA concentration and quality were estimated using a BioPhotometer Plus spectrophotometer (Eppendorf, Hamburg, Germany). Reverse transcription was performed using TakaRa PrimeScript RT Reagent Kit (Dalian, China).

Primers for amplification of 16 genes were designed using Primer Premier 5.0 and are listed in **Supplementary Table S1**. qRT-PCR was performed using a TaKaRa SYBR PrimeScript RT-PCR Kit (Dalian, China), according to the manufacturer’s instructions. The cucumber actin gene was used as an internal standard and amplified with the following primers: forward: 5′–TCGTGCTGGATTCTGGTG–3′, and reverse: 5′–GGCAGT GGTGGTGAACAT–3′. Reactions were performed in 96-well plates and the PCR program consisted of 95°C for 30 s, followed by 40 cycles of 95°C for 5 s and 50–60°C for 30 s. qRT-PCR analysis was performed on an iQ 5 multicolor real-time PCR detection system (Bio-Rad, USA). The relative expression levels of the genes were determined using the 2^-ΔΔCt^ method.

## Results

### Analysis of Numbers of Aphids per Plant

As expected, EP6392 and JY30 plants differed greatly in their resistance to aphids; EP6392 showed significant resistance to aphid infestation, whereas JY30 was susceptible. Phenotypic manifestations of this included the fact that the leaves of infested EP6392 plants curled upward slightly, whereas those of infested JY30 plants were more obviously curled upward. Moreover, EP6392 plants grew and developed normally, whereas JY30 plants were severely stunted on day 8 after aphid infestation. The F_2_ population consisted of both susceptible and resistant plants (**Table 1**); consistent with the results of our previous study (Liang et al., 2015).

**Table 1:** The frequency distribution of aphid numbers on individual EP6392, JY30, and F_2_ seedlings on day 8 after infestation.

Generation	Number	Aphid score				
		
		1	2	3	4	5
JY30 (P_1_)	27	-	-	-	10	17
EP6392 (P_2_)	28	15	13	-	-	-
F_2_	1000	180	531	200	77	12


When EP6392 plants were infested with different numbers of aphids, the average number of aphids per plant on day 6 after infestation correlated with the number of aphids used to infest the plants. The most significant difference in the number of aphids per plant was observed between T1 and T2 treatment. The average number of aphids per seedling on day 6 after aphid infestation were 80.20 and 167.20 for T1 and T2 treatment, respectively (**Table 2**).

**Table 2:** Comparison of aphid numbers among plants infested with different numbers of aphids.

Number of aphids applied	Number of aphids per plant (day 6)
5	80.20bB ± 4.13
20	167.20aA ± 4.41


*Different small letters and capital letters indicate significant difference among treatments at 0.05 and 0.01 level, respectively. The same below.*

### Analysis of SLAF-seq Data and SLAF Tags

In this study, a total of 38 million sequence data reads were obtained, each of 80 bp (×2). The majority of bases (80.55%) were of high quality (scores of ≥30; **Table 3**). The number of SLAF sequences were 99,180, and 98,812 for JY30 and EP6392 plants, respectively. Average SLAF sequence depths were 18.13-fold in the parental samples and 45.83-fold and 48.44-fold in the R- and S-pools, respectively (**Table 3**). SLAF tags (*n* = 102,033) were divided into three categories: polymorphic, non-polymorphic and repeat, according to analysis of allele frequencies and gene sequences differences. Polymorphic SLAF tags were identified (*n* = 5,471), indicating a polymorphism rate of 5.36% (**Table 4**).

**Table 3:** Summary of sequencing data.

Sample	Total reads	Q30 (%)	GC (%)	SLAF number	Total depth	Average depth
JY30 (P_1_)	5,353,262	80.56	40.88	99,180	1,808,312	18.23
EP6392 (P_2_)	5,164,837	80.79	40.35	98,812	1,781,881	18.03
R-pool	13,223,930	80.71	40.49	101,956	4,672,486	45.83
S-pool	14,259,464	80.12	40.15	101,950	4,938,765	48.44


**Table 4:** Distribution on chromosomes of SLAF tags and polymorphic SLAF tags.

Chromosome ID	SLAF tags	Polymorphic SLAF	Selected for QTL mapping
Chr1	15,613	604	235
Chr2	11,896	555	168
Chr3	20,907	1,213	454
Chr4	12,619	522	121
Chr5	14,977	1,031	373
Chr6	15,579	971	437
Chr7	10,442	575	173
**Total**	102,033	5,471	1,961


### Association Analysis

**FIGURE 1:**
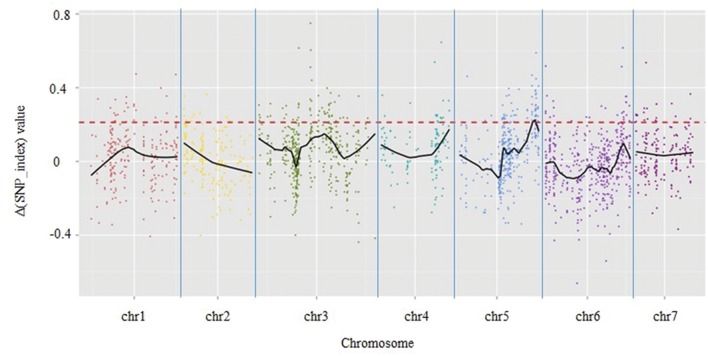
**Results of genetic association analysis to identify regions of the cucumber genome associated with resistance to aphid infestation.** X-axis = chromosome position; y-axis = Δ (SNP_index) values. Black lines are average Δ (SNP-index) values, as determined by sliding window analysis. The red dotted line is the threshold value (0.2122), which was calculated by Loess regression. Peak regions were defined as those where Loess fitted values were greater than the threshold value and one region on chromosome 5 containing 20 SLAF markers within a 0.31-Mbp-long section of the cucumber reference genome (based on the genome sequence of line 9930) was identified.

A total of 1,961 tags from among the 5,471 polymorphic SLAF tags were selected for QTL identification based on criteria of sequence depth >5-fold in parents and the genotype of one allele being derived from JY30 and the other from EP6392 (**Table 4**). Peak regions above the threshold value were defined as those where Loess fitted values were greater than standard deviations above the genome-wide median in the Δ (SNP_index) plot. One candidate region associated with cucumber aphid resistance spanning 0.31 Mb on Chromosome 5 (Chr5: 26,684,572–26,994,642, cucumber line 9930 reference genome assembly, version 2) was identified with average Δ (SNP_index) values above the threshold value of 0.2122 (**Figure 1**). The candidate region contained 43 genes and 20 SLAF makers. Based on functional annotation, 16 of the 43 genes were selected as good candidates for association with aphid resistance in cucumber (**Table 5**). Details of the 20 SLAF makers are provided in **Supplementary Table S2**.

**Table 5:** Annotation of candidate genes for aphid resistance in the QTL identified on cucumber chromosome 5.

Gene_ID	Annotation	Database
Csa5M641610.1	Transcription factor MYB59-like	Swissprot, KEGG, COG
Csa5M641620.1	Probable E3 ubiquitin-protein ligase HERC4-like	Swissprot, KEGG
Csa5M642120.1	E3 ubiquitin-protein ligase At3g02290-like	Swissprot, KEGG, COG
Csa5M642140.1	Auxin transport protein BIG-like	Swissprot, KEGG, COG
Csa5M642160.1	F-box/kelch-repeat protein At5g15710-like	Swissprot, KEGG, COG
Csa5M642710.1	Transcription factor HBP-1a-like	Swissprot, KEGG
Csa5M643280.1	F-box family protein	COG, GO
Csa5M643290.1	F-box/kelch-repeat protein	Swissprot, KEGG, COG
Csa5M643380.1	Beta-glucan-binding protein	KEGG, GO
Csa5M643880.1	Endo-1,3(4)-beta-glucanase 1-like	Swissprot, KEGG
Csa5M642150.1	Probable leucine-rich repeat (LRR) receptor-like serine/threonine-protein kinase At5g15730-like	Swissprot, KEGG, COG
Csa5M642730.1	Protein NSP-interacting kinase 1-like	Swissprot, KEGG, COG
Csa5M643240.1	Stress-induced protein KIN2	COG
Csa5M643260.1	Stress-induced protein KIN2	COG
Csa5M643350.1	Glycine-rich cell wall structural protein 1.0	COG
Csa5M643900.1	Proline-rich receptor-like protein kinase PERK10-like	Swissprot, KEGG, COG


### Influence of Aphid Infestation on Expression of Candidate Genes

Analysis of gene expression levels by qRT-PCR revealed that levels of seven genes, encoding the transcription factor MYB59-like (Csa5M641610.1), auxin transport protein BIG-like (Csa5M642140.1), F-box/kelch-repeat protein At5g15710-like (Csa5M642160.1), transcription factor HBP-1a-like (Csa5M6-42710.1), beta-glucan-binding protein (Csa5M643380.1), endo-1,3(4)-beta-glucanase 1-like (Csa5M643880.1) and proline-rich receptor-like protein kinase PERK10-like (Csa5M643900.1), were best significantly lower in T1 and T2 treatments than in CK, indicating that these genes were down-regulated after aphid infestation.

Conversely, the expression levels of four genes, encoding stress-induced protein KIN2 (Csa5M643240.1 and Csa5M643260.1), probable LRR receptor-like serine/threonine-protein kinase At5g15730-like (Csa5M642150.1) and F-box family protein (Csa5M643280.1) were significantly higher in T1 and T2 treatments than in CK. Moreover, the expression levels of Csa5M643240.1 and Csa5M643260.1 were best significantly higher in T2 treatment than in T1 treatment. The expression level of the gene encoding F-box family protein (Csa5M643280.1) in T2 treatment was best significantly higher than in CK, and was higher than in T1 treatment. The expression level of the gene encoding F-box/kelch-repeat protein (Csa5M643290.1) in T2 treatment was best significantly higher than in T1 treatment and in CK, and expression of this gene level was higher in T1 treatment than in CK; however, the difference was not statistically significant. Hence, these five genes were up-regulated after aphid infestation.

**FIGURE 2:**
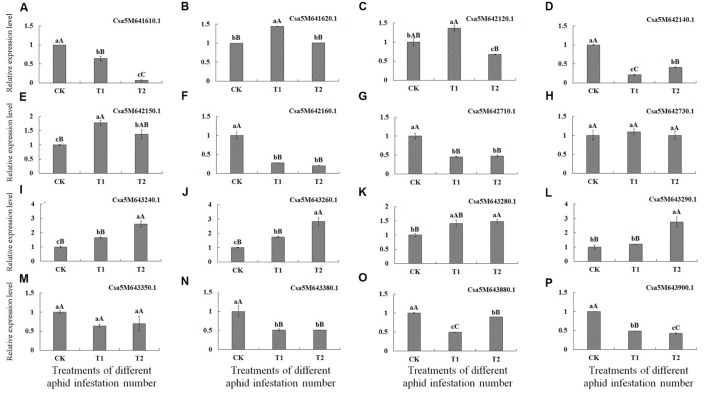
**Expression of the 16 genes identified by SLAF-seq after infestation of cucumber plants with different numbers of aphids determined by qRT-PCR.** X-axis = number of aphids applied, CK = 0 (control), T1 (5 aphids), T2 (20 aphids). Y-axis = relative expression level. **(A–P)** Represent the relative expression level of 16 genes after infestation with different numbers of aphids in cucumber plants determined by qRT-PCR, respectively.

The expression of genes encoding probable E3 ubiquitin-protein ligase HERC4-like (Csa5M641620.1) and E3 ubiquitin-protein ligase At3g02290-like (Csa5M642120.1) did not appear to be regulated in response to aphid infestation. In addition, the expression levels of genes encoding protein NSP-interacting kinase 1-like (Csa5M642730.1) and Glycine-rich cell wall structural protein 1.0 (Csa5M643350.1) in T1 and T2 treatments were not significantly different from those in CK (**Table 6**; **Figure 2**).

**Table 6:** Regulation of candidate genes in response to aphid infestation in cucumber.

Gene_ID	Annotation	Up- regulated	Down- regulated	Note
Csa5M641610.1	Transcription factor MYB59-like		√	The expression level was very low
Csa5M642140.1	Auxin transport protein BIG-like		√	
Csa5M642150.1	Probable LRR receptor-like serine/threonine-protein kinase At5g15730-like	√		
Csa5M642160.1	F-box/kelch-repeat protein At5g15710-like		√	
Csa5M642710.1	Transcription factor HBP-1a-like		√	
Csa5M643240.1	Stress-induced protein KIN2	√		The expression level was very low
Csa5M643260.1	Stress-induced protein KIN2	√		The expression level was very low
Csa5M643280.1	F-box family protein	√		The expression level was very high
Csa5M643290.1	F-box/kelch-repeat protein	√		The expression level was very low
Csa5M643380.1	Beta-glucan-binding protein		√	The expression level was very high
Csa5M643880.1	Endo-1,3(4)-beta-glucanase 1-like		√	
Csa5M643900.1	Proline-rich receptor-like protein kinase PERK10-like		√	


## Discussion

Aphid is one of major pests affecting cucumber production. Severe aphid infestation can cause many visible symptoms, including leaf curling and yellowing, and plant wilting and stunting. In another aspect, the virus propagate with extreme rapidity by aphid. Understanding of the genetic mechanisms underlying aphid resistance is the most effective way to decrease aphid damage and produce improved quality cucumbers. SLAF-seq technology is an effective and high-resolution technique for fine mapping of QTLs. The calculation of the SNP-index allows accurate quantitative evaluation of the frequencies of parental alleles, as well as the genomic contribution from the two parents, in F_2_ individuals. The combination of SLAF-seq technology, the SNP-index method and BSA provides an efficient method to identify genomic regions related to traits. In this study, we used this approach to analyze two pooled F_2_ population samples and detect a genomic region associated with aphid resistance in cucumber. A main effect QTL of 0.31 Mb on Chr5, including 43 genes, was obtained by association analysis. Sixteen of the 43 genes were identified as candidates for association with aphid resistance based on gene annotation.

Plant hormones have important roles in signaling, and are vital in plant defenses against pathogens (Pieterse et al., 2009). The expression levels of a gene encoding the auxin transport protein BIG-like, was down-regulated in cucumber plants in response to aphid infestation. When green peach aphid (*Myzus persicae* Sulzer) infest *Arabidopsis thaliana*, the synthetics of Salicylic acid (SA) is promoted (Pegadaraju et al., 2005). Moreover, sugars are important in the defense of plants against pest infestation. Genes associated with sugar metabolism in *Apium graveolens*, *Arabidopsis thaliana* and *Nicotiana attenuate* are up-regulated after aphid infestation (Moran et al., 2002; Heidel and Baldwin, 2004; Voelckel et al., 2004; Divol et al., 2005; Qubbaj et al., 2005; Thompson and Goggin, 2006). However, in this study, the expression level of the gene encoding endo-1,3(4)-beta-glucanase 1-like was down-regulated in cucumber plants in response to infestation, which differs to the findings of previous studies in other plant species. Genes encoding the transcription factors MYB59-like, the transcription factor HBP-1a-like, the F-box/kelch-repeat protein At5g15710-like, the beta-glucan-binding protein and the proline-rich receptor-like protein kinase PERK10-like were also down-regulated in plants infested with different numbers of aphids. Our results indicate that aphid infestation resulted in repression of these seven genes and that they may be associated with aphid resistance in cucumber (**Table 6**).

E3 ubiquitin ligase can function as a regulator of plant disease-resistance signaling in *Arabidopsis thaliana* (Stegmann et al., 2012), rice (Park et al., 2012), and tobacco (Gilroy et al., 2011). In a study revealing the mechanism of interaction between the rice blast fungus effector factors AvrPiz-t and relative R gene Piz-t (Park et al., 2012) found that APIP6, which interacts with the effector AvrPiz-t, is a RING type E3 ubiquitin ligase, which positively influences the immune response to rice blast fungus. Stegmann et al., (2012) found that E3 ubiquitin ligase can negatively regulate immune responses induced by pathogen associated molecular patterns (PAMPs). In this study, the expression levels of genes encoding the probable E3 ubiquitin-protein ligase HERC4-like and E3 ubiquitin-protein ligase At3g02290-like, were not obviously regulated in plants infested with different numbers of aphids, inconsistent with previous reports in other plant species.

R genes with LRR domains have major roles in regulation of the resistance of plants to pathogens and insects. Such genes confer resistance to the blue alfalfa aphid in *Medicago truncatula* Gaert (Klingler et al., 2005) and to soybean aphid in soybean (Kim et al., 2010a,b). The expression level of the gene Csa5M642150.1, encoding probable LRR receptor-like serine/threonine-protein kinase At5g15730-like, was up-regulated in cucumber plants in response to infestation with aphids. The expression levels of the genes encoding F-box family protein, F-box/kelch-repeat protein and the genes (Csa5M643240.1 and Csa5M643260.1) encoding stress-induced protein KIN2 were also up-regulated in plants in response to aphid infestation. These results indicate that aphid infestation enhanced expression of these five genes, suggesting that they could play an important role in aphid resistance in cucumber.

## Author Contributions

XC, FZ, XQ, and DL conceived the experiment and made the revision of the manuscript. DL and MC performed the research. DL and MC collected data. DL, MC, and QX analyzed the date and wrote the manuscript. All authors reviewed and approved this submission.

## Conflict of Interest Statement

The authors declare that the research was conducted in the absence of any commercial or financial relationships that could be construed as a potential conflict of interest.
