# Supplementary material for: QTL Mapping by SLAF-seq and Expression Analysis of Candidate Genes for Aphid Resistance in Cucumber
Source: Front Plant Sci. 2016 Jul 11;7:1000. doi: 10.3389/fpls.2016.01000 (PMC4939294; doi:10.3389/fpls.2016.01000)
Supplement: Supplementary file 1 [file Table_1.DOCX]

# Supplementary Table S1. Primers used for qRT-PCR

| Gene_ID | Forward primer(5’-3‘) | Reverse primer(5’-3‘) | Product(bp) |
| --- | --- | --- | --- |
| Csa5M641610.1 | ATACCCATTTGGCATCCA | TTCCTCCATTCTTGCTCTTC | 90 |
| Csa5M641620.1 | TTCCGAAGGTCTACAAGG | AAGCGAACAGTGACCAAG | 184 |
| Csa5M642120.1 | TGTACCGCTCACCTCCAA | AGCAGACCCATTCCGTTT | 191 |
| Csa5M642140.1 | CTTCCCTCGGTCTTTCAC | AGCATCAATCGCCCTAAT | 114 |
| Csa5M642150.1 | GGAGGAATCATCACCACA | AAGCGAGCACCTTAACAG | 180 |
| Csa5M642160.1 | AACAAGTTTCGCCTCTAA | GACCGTTTCCTCATCTAA | 131 |
| Csa5M642710.1 | GGCACAGAACAACTTACC | AAACACGGCTGTCTAAAA | 102 |
| Csa5M642730.1 | AAGAACATACTAGGAAAGGGAG | CTGAGGAGATGACGGTGC | 170 |
| Csa5M643240.1 | GGCAGACAATAACTCTAACAA | TCGCCTTATCCATCAAAT | 92 |
| Csa5M643260.1 | CTGGAGAAGCCAAAGGTC | CCTGCCTCCTGTATCGTC | 100 |
| Csa5M643280.1 | CTCTTGCCATGCTCTACT | ATCCCTTTCGGTAACTCT | 193 |
| Csa5M643290.1 | TGGAGGTTATGAGGTTTG | CGTGTTGTAAGATGGGTC | 218 |
| Csa5M643350.1 | TATTTCTCGGTGGTTTAGC | TTCTCAGTGGCAAGTTTCT | 298 |
| Csa5M643380.1 | CTTAATAATGGCGACCAA | ACGAGATAGAAACGGAGG | 251 |
| Csa5M643880.1 | TTGGGTTTGGCTTATGGA | TCTCAGTGATGGGCAGTA | 256 |
| Csa5M643900.1 | GCTTGCGTTCGTCATTCG | GCCCAGGTTTCACTCCATT | 109 |
