# Supplementary material for: QTL Mapping by SLAF-seq and Expression Analysis of Candidate Genes for Aphid Resistance in Cucumber
Source: Front Plant Sci. 2016 Jul 11;7:1000. doi: 10.3389/fpls.2016.01000 (PMC4939294; doi:10.3389/fpls.2016.01000)
Supplement: Supplementary file 2 [file Table_2.DOCX]

# Supplementary Table S2. SLAF makers significantly associated with aphid resistance

| SLAF_ID | Chr_ID | Start | End | Smooth |
| --- | --- | --- | --- | --- |
| Marker237124 | Chr5 | 26684572 | 26684797 | 0.2246 |
| Marker233138 | Chr5 | 26725364 | 26725598 | 0.2232 |
| Marker244668 | Chr5 | 26728098 | 26728299 | 0.2231 |
| Marker243373 | Chr5 | 26728522 | 26728868 | 0.2230 |
| Marker223014 | Chr5 | 26734051 | 26734324 | 0.2228 |
| Marker221858 | Chr5 | 26736753 | 26736989 | 0.2227 |
| Marker234766 | Chr5 | 26737869 | 26738201 | 0.2227 |
| Marker218872 | Chr5 | 26775373 | 26775656 | 0.2213 |
| Marker251487 | Chr5 | 26794364 | 26794609 | 0.2206 |
| Marker237992 | Chr5 | 26831180 | 26831357 | 0.2191 |
| Marker246198 | Chr5 | 26857767 | 26858120 | 0.2181 |
| Marker240720 | Chr5 | 26866153 | 26866348 | 0.2177 |
| Marker226458 | Chr5 | 26919001 | 26919270 | 0.2155 |
| Marker204371 | Chr5 | 26927340 | 26927539 | 0.2152 |
| Marker222152 | Chr5 | 26928852 | 26929109 | 0.2151 |
| Marker228165 | Chr5 | 26930724 | 26931052 | 0.2151 |
| Marker218209 | Chr5 | 26961355 | 26961639 | 0.2138 |
| Marker242745 | Chr5 | 26975716 | 26976059 | 0.2131 |
| Marker211644 | Chr5 | 26980020 | 26980326 | 0.2130 |
| Marker216256 | Chr5 | 26994311 | 26994642 | 0.2124 |
